# Has maternal sensitivity been comprehensively investigated in sub-Saharan Africa? A narrative scoping review

**DOI:** 10.1017/neu.2023.20

**Published:** 2023-03-24

**Authors:** A. Prag, K.A. Donald, E. Weldon, S.L. Halligan, D.J. Stein, S. Malcolm-Smith

**Affiliations:** 1 ACSENT Laboratory, Department of Psychology, University of Cape Town, Cape Town, South Africa; 2 Neuroscience Institute, University of Cape Town, Cape Town, South Africa; 3 Division of Developmental Paediatrics, Department of Paediatrics and Child Health, Red Cross War Memorial Children’s Hospital, University of Cape Town, Cape Town, South Africa; 4 Department of Psychology Bath University, England; 5 Department of Psychiatry and Neuroscience Institute, Groote Schuur Hospital, University of Cape Town, Cape Town, South Africa; 6 MRC Unit on Risk & Resilience in Mental Disorders, Cape Town, South Africa

**Keywords:** maternal sensitivity, risks, outcomes, cross cultural measure, Sub-Saharan Africa, observational study

## Abstract

Child development is strongly influenced by maternal characteristics. Maternal sensitivity, as well as risks to and outcomes of sensitive maternal style, are well studied in industrialised western contexts, but it is unclear if this is the case for other contexts. Sub-Saharan Africa has been subjected to and continues to negotiate socio-economic and psychological sequelae of colonial and race-based politics: exploring the nature and outcomes of early caregiver input in such challenging conditions is imperative. This scoping review thus aims to 1) evaluate the nature and extent of quantified observational assessments of dyadic interactions, with a focus on maternal sensitivity, in Sub-Saharan Africa and 2) ascertain which risk and outcome factors have been examined in relation to maternal sensitivity. Study quality and cross-cultural appropriateness will also be considered. The search using expanded search terms yielded 20 papers –four characterizing maternal sensitivity or style, eight examining maternal sensitivity in relation to risks and outcomes, and eight intervention studies examining efforts to improve maternal sensitivity. Most research was conducted in South Africa – only seven studies were conducted in four other countries. Researchers used a wide array of coding schemes, mostly developed in the west. Ten studies made some adaptations to measures. Language issues and cultural considerations were often not explicitly addressed. Taken together, very limited research on this important topic exists. For the work that does exist, questions around westernized assumptions, language, and appropriateness of measures remain. Substantially more research, informed by both culturally flexible conceptualizations and methodological rigour, is required.

## Summations

- A search using all the core foci search terms yielded zero papers; therefore, a single standard PRISMA compliant search method could not be used. Six searches were conducted (Ebscohost – Academic Search Premier, MEDLINE, APA PsychArticles, APA PsychInfo, and APA PsychTests) using various combinations of the core foci for a yield of relevant research data.
- The reviewed research shows some findings consistent with research from HICs. However, this small set of studies from a limited number of contexts cannot be considered to provide a full articulation of the nature of maternal style or sensitive caregiving for sub-Saharan Africa, let alone factors promoting or disrupting it, or the child developmental outcomes associated with variability in caregiving.
- More research is needed to understand the emerging child influenced by maternal sensitivity, child temperament, along with cultural and environmental factors, to foster optimally targeted interventions for mothers and children in low- to middle-income countries contexts, and to avoid biased interpretation of behaviours occurring in non-western environments.

## Considerations

- Maternal sensitivity was not defined or operationalized uniformly enough to allow for a meta-analysis to be conducted.
- The heterogeneity in research foci and methods also made descriptive comparisons across studies difficult.
- A hopeful sign is that a number of recently published papers have focused on maternal sensitivity in LMICs – suggesting that researchers are recognising the need to address the dearth of knowledge on the fundamental functioning and ongoing impact of this phenomenon in non-western contexts.

## Introduction

Parenting practices vary across countries, socio-economic circumstances, parental psychological resources, child characteristics, and contextual sources of stress and support (Fish, 2001; Whittaker *et al*., 2011). At present, most theorising and empirical findings regarding maternal style and characterising the nature of the primary caregiver–infant dyad seem to be found in relation to North American, Australian and European samples (Mesman, Minter, *et al*., 2018; Mesman *et al*., 2012), where it is widely understood that improving the mother (primary caregiver)–child relationship can have significantly positive effects on child development (Bakermans-Kranenburg *et al*., 2003; Joosen *et al*., 2012). In an African context of growing dissatisfaction with imported ideas and knowledge and increased questioning of the validity of such knowledge (Kiguwa & Segalo, 2018), it is necessary to delineate and interrogate the knowledge base that exists for African dyads in the psychological literature. Many have argued that a more critical approach to psychology research in sub-Saharan Africa, challenging in particular neo-colonial ideas of individualism, can better guide the field in terms of understanding and responding to the mental health challenges created in large part by historically colonial economic, social, and political contexts (Cullen *et al*., 2021). Any unquestioning adoption of western methods and constructs is considered inappropriate for contexts that have divergent conceptualisations of what it means to be a psychological being (Kiguwa & Segalo, 2018; Seehawer, 2018) and of parenting skills and goals within a more collectivistic society (Seehawer, 2018). We thus need to carefully consider the evidence for potentially universal principles regarding sensitive caregiving and its outcomes and examine the methods and models that underlie such findings as well as those that diverge from accepted norms.

Research from low- to middle-income countries (LMICs) reported in a series of reviews exploring inequality in early childhood proposes that a conservative estimate of 200 million children under the age of 5 fail to reach their potential in development due to poverty, poor health and nutrition, and inadequate care (Grantham-McGregor *et al*., 2007; Walker *et al*., 2007; Walker *et al*., 2011). In sub-Saharan Africa, a long history of colonial influence has had deleterious consequences on its citizens, often further exacerbated by subsequent mal-administration and high levels of internal conflict (Luiz, 2006, 2013). Social welfare systems and psychological health development are regarded as having struggled to evolve from imported colonial ideologies and have failed to serve the needs of disenfranchised citizens (Nsamenang & Dawes, 1998; Luiz, 2006; Nsamenang & Dawes, 1998). South Africa, for example, has one of the highest Gini coefficients’ (0.65 in 2015 – Galal, 2021) indicating vast economic disparities between communities. This is known to drive high levels of violence, crime, and specifically intimate partner violence (Yapp & Pickett, 2019), while social support for citizens who have experienced the worst consequences is limited (Maluleke, 2018). Existing research has identified multiple risk factors in LMICs that negatively impact child development, including, *inter alia*, maternal depression, inadequate cognitive stimulation, and exposure to societal violence (Grantham-McGregor *et al*., 2007; Walker *et al*., 2007; Walker *et al*., 2011). Among the identified risk factors, several are known to affect the kind of care the child receives, and/or that the mother is able to provide.

In socio-economic contexts, where health and community resources are scarce, individual differences in a broader framework of maternal characteristics would constitute a central factor in determining the child’s adequate physical growth, cognitive, and socio-emotional development (Fraley *et al*., 2013; Harris *et al*., 2014; Harmeyer *et al*., 2016). However, maternal competence/style (including sensitivity) may be adversely affected in challenging contexts (Mesman *et al*., 2012; Hosokawa & Katsura, 2017). For example, the potential negative effect maternal depression has on a child’s socio-emotional development – mediated through maternal sensitivity – is well documented in high-income countries (Murray & Cooper, 1997; Trapolini *et al*., 2008; Gummerum *et al*., 2010; Gelaye *et al*., 2016).

Maternal sensitivity has been shown to be central in creating a secure mother–child bond, and critically development of this construct was based on research conducted in sub-Saharan Africa (Uganda) and the USA. Mary Ainsworth’s definition of a sensitive mother obtained through her seminal research included three components – 1) observation of child cues; 2) correct perception and interpretation of child cues, and 3) appropriate behavioural and emotional responses to child cues (Ainsworth *et al*., 1978). Sensitive responsiveness has been shown to cultivate a secure and trusting relationship, with caregivers being experienced as available and reliable. Maternal sensitivity is understood to encompass the attuned (timely, contingent, and appropriate) manner in which mother as primary caregiver attends to her child (Murray *et al*., 1996; Whittaker *et al*., 2011), keeping in mind individual temperament and other nuances of her child’s needs. Parental characteristics, such as sensitivity, including the ability to scaffold and structure activities, along with a lack of hostility and intrusiveness in particular, are considered important in creating an emotionally open and available relationship with the child (Biringen & Robinson, 1991; Bornstein & Manian, 2013; Biringen *et al*., 2014). Furthermore, accurate identification of negative infant emotions, emotional responses to distress, parenting goals, and emotional efficacy (inclusive or exclusive of warmth) influence the quality of dyadic interactions (Lohaus *et al*., 2001).

Since the original maternal sensitivity construct emerged as pivotal to understanding the nature of parent–child relationships, non-gendered descriptive terms such as “parental sensitivity” have been introduced into the literature in HICs (Broom, 1994) and has been incorporated by experts in the field (Bakermans-Kranenburg *et al*., 2003; Mesman *et al*., 2012). However, because most early research has focussed on the mother–infant bond (Cooke *et al*., 2022), we elected to focus on this original terminology.

Maternal warmth has been understood to entail an expression of either smiling or empathy during positive and negative infant emotions, respectively. Talking to the infant in engaging age-appropriate tones and language is also considered a component of warmth (Lohaus *et al*., 2001). Since Mary Ainsworth’s Sensitivity/Insensitivity Scale, which excluded overt expressions of warmth, several sensitivity instruments developed since then have included aspects of warmth and variations of positive maternal affect as integral components of the sensitivity construct. In a review of sensitivity observational instruments used since Ainsworth’s scale, Mesman and Emmen found that of the eight main instruments, seven included warmth/positive affect (or a variation of the construct) as a significant component in measuring maternal sensitivity. Studies of the role warmth plays in maternal sensitivity have yielded varied results almost exclusively from samples in the global north (Mesman & Emmen, 2013). Rural samples in sub-Saharan Africa have in the past been misunderstood to be insensitive in part due to their lack of overtly expressed warmth (Kermoian & Leidermann, 1986). The role of warmth in characterising maternal sensitivity should be examined in sub-Saharan African and possibly other LMIC research.

The relationship between maternal sensitivity and child outcomes continues to be a relevant avenue of research. A recent global review of maternal sensitivity and its impact on child outcomes (Deans, 2018) highlighted several domains of functioning directly (*k* = 46) influenced by maternal sensitivity. The areas tested were varied but included cognitive (language acquisition, general cognitive and executive functioning, attentional control) and physical factors (obesity, sleep). Broadly, socio-emotional factors included – behavioural problems, social competence, emotionality, and temperament. The precise characteristics investigated within each domain were numerous and heterogenous.

Deans noted a caveat – great heterogeneity in study methods and the definition of maternal sensitivity informed a broad summary rather than a detailed analysis of studies reviewed. All of the domains of functioning directly impacted by maternal sensitivity represented in this list were researched in a HIC context.

The extent to which maternal sensitivity directly impacts or mediates various child outcomes in sub-Saharan Africa as has been demonstrated in HIC’s is unclear. Socio-economic disparities can have a synergistic influence on what takes place in parenting and child outcomes (Walker *et al*., 2007; Walker *et al*., 2011). Varying levels of socio-economic status within a region could further inform developmental expectations where middle class and affluent families that make up a smaller part of the region’s population may be more acculturated to western norms (Grantham-McGregor *et al*., 2007; Walker *et al*., 2011). In a country such as South Africa, where majority of households are economically disadvantaged (*Stats SA General Household Survey*, 2021), different risks or contexts to those found in HIC’s could apply. The implications of stressful factors such as compromised maternal mental health and socio-emotional competence, limited social and economic support, inadequate living conditions (intense daily threats to personal safety), substance abuse and community, and personal trauma interacting with various child characteristics are fairly unclear. Comprehensive evidence of what these factors are in sub-Saharan Africa and how they impact child development is not yet known.

Following Mary Ainsworth’s original Ugandan research, there appears to be a gap in the knowledge production from sub-Saharan Africa as compared to elsewhere in the world based on the fundamental principles of primary caregiver/s and sensitivity. Therefore, the aims of this review are 1) to scope and clarify precisely what quantitative, objectively coded research has been conducted on maternal sensitivity in Sub-Saharan Africa and 2) to identify which risk factors and child outcomes related to maternal sensitivity have been investigated in this region. The central question of the quality and appropriateness of methods will be considered in reporting the results.

## Methods

### Search method

The search aimed to find research on observational studies of maternal sensitivity. The decision to review research that employed videoed observed dyadic interactions was made due to its relative methodological strength as compared to maternal self-report measures. Observed interactions allow for objectivity in coding that can be verified by a team of coders, reducing bias in both the participant (social desirability bias in self-report) and observer/coder (Althubaiti, 2016). Considering the synergistic nature of maternal characteristics and child development, we also included a search of risks and outcomes of maternal sensitivity assessed in such observational studies.

The first author and a subject librarian at the University of Cape Town set up key concept search terms and included terms using the expanded thesaurus function for the databases on Ebscohost – Academic Search Premier, MEDLINE, APA PsychArticles, APA PsychInfo, and APA PsychTests (see search terms in Table 1).

**Table 1.** Search terms

Core Concepts	Expanded Terms
Maternal sensitivity (main core search)	**“Maternal sensitivity”**
Videoed interactions (main core search)	**Observed OR Recorded OR Videoed OR Video OR Recording OR Record OR “Observational studies”**
Dyad	**DE “mother-child relationship” OR “dyadic interactions” OR “mother-infant” OR “mother-toddler” OR “maternal dyad” OR “mother-child”**
Risks in relation to maternal sensitivity	**“Risk factors” OR DE “Sociocultural Factors” OR DE “Psychosocial Factors” OR DE “Risk Factors” OR DE “mental health” OR DE “Mental Status” OR DE “Physical Health”**
Outcomes in relation to maternal sensitivity	**Child Outcomes OR “Physical Health” OR DE “Psychological Development” OR DE “Physical Development” OR DE “Socioemotional Functioning” OR DE” Psychosocial Development” OR DE “Cognitive Ability” OR DE “Child Characteristics” OR DE “Early childhood development”**
sub-Sahara Africa (main core search)	**Angola OR Benin OR Botswana OR “Burkina Faso” OR Burundi OR “Cabo Verde” OR Cameroon OR Cameroun OR “Canary Islands” OR “Cape Verde” OR “Central Africa” OR “Central African Republic” OR Chad OR Comoros OR Congo OR “Cote d'Ivoire” OR “Democratic Republic of Congo” OR Djibouti OR “Eastern Africa” OR Eritrea OR eSwatini OR Ethiopia OR Gabon OR Gambia OR Ghana OR Guinea OR “Guinea-Bissau” OR “Ivory Coast” OR Jamahiriya OR Kenya OR Lesotho OR Liberia OR Madagascar OR Malawi OR Mali OR Mauritania OR Mauritius OR Mayotte OR Mozambique OR Namibia OR Niger OR Nigeria OR Principe OR Reunion OR Rwanda OR “Sao Tome” OR Senegal OR Seychelles OR “Sierra Leone” OR “Saint Helena” OR Somalia OR “St Helena” OR “South Africa” OR “Southern Africa” OR Sudan OR Swaziland OR Tanzania OR Togo OR Uganda OR “ Western Africa” OR “Western Sahara” OR Zaire OR Zambia OR Zimbabwe**

#### Inclusion criteria

To be included, articles needed at minimum to include research on observed videoed and coded maternal sensitivity or style for dyads in sub-Saharan Africa. Only English language, published, peer-reviewed papers were included. No date restriction was applied.

This review initially aimed to determine the extent of data available addressing the full combination of research foci. However, conducting a full key concept search (maternal sensitivity, videoed interactions, dyad, risks to maternal sensitivity, outcomes of maternal sensitivity, sub-Saharan Africa), yielded one article which did not fulfil inclusion criteria, therefore several subset concept searches were required (see Table 1).

**Fig. 1:**
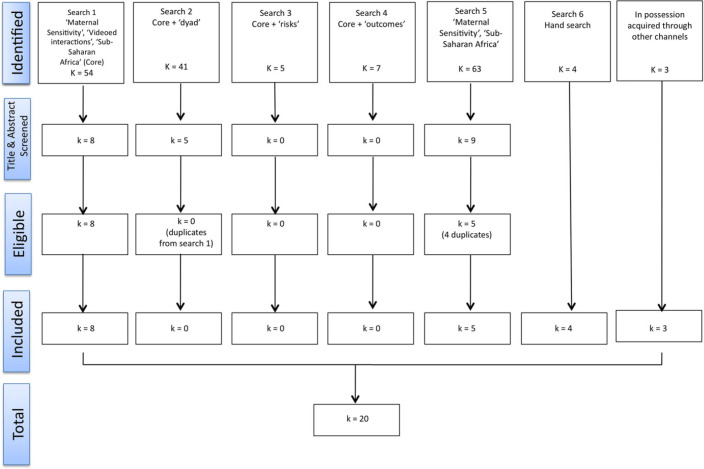
Search Flow Diagram.

Six separate searches across databases were conducted – see Fig. 1 below. Initially, search 1 yielded *k* = 54 papers, search 2: *k* = 41; search 3: *k* = 5; search 4: *k* = 7 and search 5: *k* = 63. Titles and abstracts were screened and *k* = 22 remained. Of these, only *k* = 13 were eligible for inclusion due to article duplication across search results. We hand-searched the reference lists of those that met inclusion criteria and found *k* = 4 additional papers. The first author had in her possession three articles (Klein and Rye, 2004; Broesch *et al*., 2016; Murray *et al.*, 2016) which were not found in the search process. Broesch *et al*. (2016) had no key words listed. As these are of significance to this review, they were included. To be thorough, a separate search using the key search term listed in the remaining two papers “parent child interaction” with the countries was conducted. These two papers were then found. Importantly, this additional search/search term yielded no other new papers. In total, 20 articles were found.

### Evaluation of study quality

The quality of the reviewed papers was assessed in two ways: firstly via a standard empirical quality assessment tool (Downs & Black, 1998), and secondly, because this tool does not include evaluation of cross-cultural methodology, we also examined consideration of key factors in evaluating the appropriateness of such research (Aival-Naveh *et al*., 2019).

The Downs and Black methodological quality assessment tool was developed for randomised and non-randomised studies of healthcare interventions (see Table 2). The tool addresses quality of reporting, external validity (generalisability), and internal validity (bias and confounding) of the study. Each paper was rated as either “excellent” (24–28 points), “good” (19–23 points), “fair” (14–18 points), or “poor” (<14 points). This tool has been reported to be a valid, reliable, and commonly used scale (Downs & Black, 1998; O'Connor *et al.*, 2015). Given its widely accepted use and that eight papers were intervention studies, this tool was used despite its inability to capture other key aspects needing consideration in social science research.

**Table 2.** Maternal sensitivity observation coding methods and key results

Author	Region	Sample Size (Dyads) and Age	Observation Context	Maternal Sensitivity Measure	Key Results	Rating*
Maternal Sensitivity Observed						
1. Dixon, S., LeVine, R.A., Richman, A. & Berry Brazelton, T. (1984)	Kenya USA	*n* = 18 Gusii *n* = 18 Boston 6–36 months	Kenyan dyads videoed interacting during a teaching task in their home compounds. American dyads videoed in lab setting.	*No specific sensitivity measure used.* Maternal behaviour coded for presence & frequency of task modelling, setting up, simplifying, complicating, focussing or moving the child, social behaviour, and ability to attend to the child.	Maternal responsiveness to infant, an aspect of sensitivity was evaluated. Gusii mothers employed clear goal-directed instructions aimed at successful task completion mirroring cultural values of skills training. Were more physical, pulling or pushing infants which did not seem to bother them -not interpreted as insensitive. American mothers used praise and verbal encouragement to structure teaching and these infants did not respond well to any physical manoeuvring.	18
2. Broesch, T., Rochat, P., Olah, K., Broesch, J. & Henrich, J. (2016)	Fiji USA Kenya	*n* = 26 Fijians *n* = 24 US *n* = 16 Kenyans 2–12 months	Fijian and Kenyan dyads videoed interacting without touching in a room in their home or outside. American dyads did the same in a lab setting.	*No specific sensitivity measure used.* Maternal behaviours event coded across facial, vocal, gaze, and tactile modalities.	Focussed on responsiveness patterns by mothers. Mothers from all three communities had similar response rates to infants – differences emerged in selective responses to infant emotion. American and Kenyan mothers responded to positive infant facial expressions and vocalisations. Fijian mothers had more negative facial expressions and responded more to infant negative facial expressions. American mothers rested their hand on infant frequently.	20
3(a) Mesman, J., Basweti, N. & Misati, J. (2018)	Kenya	*n* = 7 6–24 months	Families were videoed for periods during the day conducting daily life activities.	Adapted Ainsworth Maternal Sensitivity scale – focus on measuring infant Received Sensitivity	4 infants had an adequate Received Sensitivity score and 3 were rated as inadequate. Sensitive responsiveness was expressed non-verbally in abundance mostly during feeding. Some insensitivity was observed during multi-tasking when other caregivers were unavailable and during bath time (a goal-orientated task to complete quickly). Sensitive responding behaviours to soothe infant were present immediately after completed tasks.	19
3(b) Mesman, J., Basweti, N. & Misati, J. (2020)	Kenya	As in 3(a) Caregiver *n* = 9	As in 3(a)	Ainsworth Maternal Sensitivity Scale Adapted Ainsworth Maternal Sensitivity Scale – focus on measuring infant Received Sensitivity by multiple caregivers. Warmth rated on scale of 1-4. One main caregiver is a father.	Three families were rated low on sensitivity, two medium and two high. Sensitivity mostly non-verbal and highest during feeding time. Non-parental caregivers were equally capable of sensitivity. Two infants were taken care of by more than one individual – overall received sensitivity evaluated for sample. Most insensitivity during hazardous domestic chores and bathing. Warmth measured as a separate variable: 4 caregivers measured low; 3 medium and 2 high. Higher warmth correlated with higher sensitivity scores.	19
Maternal Sensitivity Observed in Relation to Risks and Outcomes						
4. Cooper, P.J., Tomlinson, M., Woolgar, M. Murray, L., Molteno, C. (1999)	South Africa (Khayelitsha, Cape Town)	*n* = 147 2 months	Videoed interacting during a 5 min play period in a lab setting at a research base in Khayelitsha.	No formal name provided. Behaviours coded: general sensitivity, infant positive engagement, attentiveness & positive affect, overall quality of interaction	Maternal sensitivity was poor during infant engagement. Infants were less positively engaged during poor maternal interactions.	22
5. McMahan True, M., Pisani, L. & Oumar, F. (2001)	Mali	*n* = 42 (*n* = 27 rural *n* = 15 village) 10–12.5 months	Videoed at home including bathing and cooking episodes. Strange Situation and Weigh- In Procedure conducted outside in a makeshift lab setting.	Adapted Ainsworth Maternal Sensitivity Scale	Maternal sensitivity correlated with attachment status during bathing episode.	23
6. Perez, E., Hendricks, M.K., Beard, J.L., Murray-Kolb, L.E., Berg, A., Tomlinson, M., Irlam, J., Isaacs, W., Njengele, T., Sive, A. & Vernon-Feagans, L. (2005)	South Africa (Khayelitsha, Cape Town)	*N* = 81 IDA: *n* = 30 IDA placebo: *n* = 21 IDA control: *n* = 30 T1: 10 weeks T2: 9 months	Videoed play interactions in a lab setting.	Parent Caregiver Involvement Scale	At T1 visit mean scores of the 11 PCIS scales did not differ significantly among the control, placebo, and anaemic sample groups. Descriptively, anaemic mothers tended to respond less frequently to infant; had fewer positive and negative statements and exerted more control over infant behaviour. At T2, more significant group variances evident: placebo-treated anaemic mothers were less responsive and goal-directed and made more negative comments towards infants as compared to the control group. Iron-treated and control group mothers were similar and related to infants more positively and did not display as much of the negative mothering found in non-treated mothers.	21
7. Tomlinson, M., Cooper, P.J. & Murray, L. (2005)	South Africa (Khayelitsha, Cape Town)	As in 4 Added T2: 18 months	As in 4	T1 & T2 – Maternal behaviour rated on a general measure of sensitivity (including responsiveness, acceptance, and warmth), and presence of coercive-intrusive and remote-disengaged behaviour. Names of measures are not provided.	At T1 mothers of insecurely attached infants were significantly less sensitive, more intrusive and had more remote-disengaged behaviour compared with mothers of securely attached infants. At T2 results were similar but mothers were less remote-disengaged.	24
8. Murray- Kolb, L. & Beard, J.L. (2009)	South Africa (Khayelitsha, Cape Town)	As in 6	As in 6	Emotional Availability Scale	Iron deficient anaemic mothers were less emotionally available to infants. Infants were less responsive to these mothers.	21
9. Bozicevic, L., De Pascalis, L., Schuitmaker, N., Tomlinson, M., Cooper, P. & Murray, L. (2016)	South Africa (Stellenbosch, Khayelitsha) United Kingdom	*N* = 48 *n* = 17 Khayelitsha *n* = 15 Stellenbosch *n* = 16 UK T1: 3 months	Videoed play and other interactive engagement at home.	T1 – Global Rating Scale Maternal strategy to infant distress assessed via 20 short visits within 14 days of T1 -coded using counts of dismissal (non-effective) and acknowledgement of distress (effective)	Groups did not differ on maternal sensitivity during face-to-face interactions. Mothers from Stellenbosch and Khayelitsha differed in their response to child ER -former group used more social soothing, and the latter were more dismissive. No difference in physical soothing (Stellenbosch and Khayelitsha).	24
10a) Dawson, N., Bain, K. & Mesman, J. (2018)	South Africa (Alexandra, Gauteng)	*n* = 50 2.5–15 months	Videoed interacting in a lab setting.	Ainsworth Sensitivity Scale Maternal Behaviour Q- Sort MINI	Modest correlation between scales but differed in profile of a sensitive mother. 23 mothers scored highly on the Ainsworth scale of which only 14 scored highly on the MBQS. A discrepancy in weighted items showed that the MBQS includes separate and contextually determined constructs of cooperation and intrusiveness. Scolding, teaching, verbal responsiveness and specific behavioural manifestations of sensitivity are not explicitly specified in the Ainsworth measure allowing for more subtle expressions to be scored.	21
10b) Dawson, N., Bain, K. & Mesman, J. (2021)	As in 10a	As in 10a	As in 10a	Ainsworth Sensitivity Scale Maternal Behaviour Q-Sort MINI Maternal Behaviour Q-Sort MINI with South African adaptations	Items relating to physical manipulation, praise, attention to infant smiling, and building on the focus of infant’s attention were reassigned positive and negative weighted scores in the revised South African MBQS. Mothers expressing distress at baby’s demands were assigned more weight. Sensitivity scores remained low but a significant, yet modest correlation was found between the SA MBQS and Ainsworth scale. Mothers with low Ainsworth scores had scores close to zero on the MBQS for both the original and SA revised MBQS.	21
Intervention with Maternal Sensitivity as variable						
11. Cooper, P.J., Landman, M., Tomlinson, M., Molteno, C., Swartz, L. & Murray, L. (2002)	South Africa (Khayelitsha, Cape Town)	*N* = 64 (*n* = 32 intervention group *n* = 32 comparison group) 6 months	Videoed interactions during play and feeding in a lab setting at a research base in Khayelitsha.	No formal measure name provided: maternal sensitivity, overall quality of interaction, infant’s engagement in interaction rated on an 8-point scale derived by researchers.	A simple comparison between groups showed that intervention group mothers benefitted, demonstrating greater sensitivity and positive affect during both interactions. *No pre intervention evaluation of sensitivity between intervention group and control group.* *Maternal sensitivity in this cohort described in relation to a sample of mothers evaluated at the same time point by Cooper et al, 1999 in an adjacent area of Khayelitsha.*	24
12. Klein, P. & Rye, H. (2004)	Ethiopia Kechene community, Addis Ababa	*n* = 96 12–36 months	Videoed interactions during play, feeding and bathing episodes at home.	Observing Mediational Interaction	Post intervention group mothers were more sensitive, responsive (increased and improved interactional quality; better physical and eye contact, conversation, shared attention), and optimistic about their ability to affect their child’s development than mothers in the comparison group.	20
13. Cooper, P.J., Tomlinson, M., Swartz, L., Landman, M., Molteno, C., Stein, A., McPherson, K. & Murray, L. (2009)	South Africa (Khayelitsha, Cape Town)	Intervention T1 *n* = 170 T2 *n* = 165 T3 *n* = 165 Control group T1 *n* = 184 T2 *n* = 181 T3 *n* = 177 T1: 6, T2: 12, T3: 18 months	As in 11	T1 – PCIS T2 – Play Observation Scheme and Emotion Ratings	Intervention mothers interacted with greater sensitivity and less intrusiveness (after completion of the intervention -T1 and T2) as compared to the control group. *No pre intervention evaluation of sensitivity between intervention group and control group.* *Maternal sensitivity in this cohort described in relation to a sample of mothers evaluated at the same time point by Cooper et al., 1999 in an adjacent area of Khayelitsha.*	25
14. Bigelow, A., Littlejohn, M., Bergman, N. & McDonald, C. (2010)	South Africa Cape Town (state maternity hospital)	*n* = 12 7–9 months	Videoed interactions at home.	Maternal Behaviour Q-Sort Maternal Behaviour subscale of the NCATS (nursing child assessment teaching scale)	Amount of SSC in infants’ first 24 hrs independently accounted for maternal sensitivity on both measures, indicating that early mother–infant SSC predicted subsequent maternal sensitivity. *No pre intervention assessment of sensitivity levels noted.*	21
15. Cooper, P., Vally, Z., Cooper, H., Radford, T., Sharples, A., Tomlinson, M. & Murray, L. (2013)	South Africa (Khayelitsha, Cape Town)	book sharing: *n* = 17 comparison: *n* = 13 15–17 months	Videoed interactions during play and reading in a lab setting at a research base in Khayelitsha.	Maternal reading and toy play coding details unclear – maternal sensitivity (rated low to high 1-5), maternal facilitation in book task, mother-infant reciprocity (rated through event counts).	Improved sensitivity in mothers during book sharing as well as in play post-intervention.	23
16. Murray, L., De Pascalis, L., Tomlinson, M., Vally, Z., Dadomo, H., MacLachlan, B., Woodward, C. & Cooper, P. (2016)	South Africa (Khayelitsha, Cape Town)	book sharing: *n* = 49 control: *n* = 42 (wait list) 14–16 months	Videoed interactions during play and reading in a lab setting at a research base in Khayelitsha.	Maternal reading and toy play coding details unclear – maternal sensitivity (rated low to high 1-5), maternal facilitation in book task, pointing and naming, elaboration, mother-infant reciprocity (rated through event counts)	Intervention was successful in improving quality of interactions by increasing sensitivity to infant cues, interests and elaborations during book sharing.	25
17. Pitilas, C., Berastegui, A., Halty, A., Roderiguez, P., Kamara, M. & Mesman, J. (2020)	Sierra Leone	*n* = 43 12–72 months	Videoed play interactions in a lab setting at the intervention centre.	Ainsworth Sensitivity Scale	Younger mothers with young children benefitted the most in relation to sensitivity especially if partner support was present.	23
18. Tomlinson, M., Rabie, S., Skeen, S., Hunt, X., Murray, L. & Cooper, P. (2020)	South Africa (Khayelitsha, Cape Town)	As in 13 T1	Videoed interactions during play and feeding in a lab setting at a research base in Khayelitsha.	T1 – PCIS	Intervention mothers scored higher on synchronous interaction as compared to control group mothers. Expression of love and maternal responsiveness to infant cues was descriptively somewhat higher among intervention mothers. *Post hoc analysis, no pre-test sensitivity reported.*	25

*Note* *Downs and Black rating scale- “excellent” (24–28 points), “good”(19–23 points), “fair” (14–18 points) or “poor” (<14 points).

Therefore, given that our focus was research in sub-Saharan Africa, further evaluation was undertaken in relation to three factors pertaining to important considerations in cross-cultural research – 1) translation of measures, 2) validation of measure for context, and 3) treatment of language during maternal sensitivity video coding (i.e. coded by trained first language speakers or translated and subtitled for expert researchers/clinicians to code) (Beaton *et al*., 2000; Boer *et al*., 2018; Aival-Naveh *et al*., 2019).

## Results

Twenty articles reported observational research investigating maternal sensitivity or style within the dyad. Papers reported original research, with the exception of two articles by Judi Mesman (Dawson *et al*., 2021; Mesman *et al*., 2021) found in search 1 who re-analysed original articles (Dawson *et al*., 2018; Mesman, Basweti, *et al.*, 2018) to accommodate newer insights and a recent paper by Mark Tomlinson (Tomlinson *et al*., 2020) using data from an earlier body of work (Cooper *et al*., 2002). Numerous measures were used to assess differing components of maternal sensitivity making a meta-analysis for a systematic review impossible. There are 51 countries in sub-Saharan Africa (Anderson & Connor, 2018), yet published research on this topic was found for only five of these: single studies from Mali, Ethiopia, and Sierra Leone, 4 from Kenya, and 13 from South Africa.

Key factors and findings in relation to maternal sensitivity and risks and outcomes associated with the variable were tabulated. Three types of research studies were found – 1) papers characterising maternal sensitivity or style (*k* = 4); 2) papers examining maternal sensitivity in relation to risks and outcomes (*k* = 8); and 3) papers examining the impact of interventions to improve maternal style (*k* = 8). Table 2 provides details on the maternal sensitivity observations. Table 3 shows the measures and results of risks and outcomes assessed. Also reported in Table 3 is maternal sensitivity as a variable for intervention in relation to risks and outcomes.

**Table 3.** Risks and outcome measures and results

Author	Measure	Risks/ Outcomes	Result
Maternal Sensitivity Observed in Relation to Risks and Outcomes			
4. Cooper, P.J., Tomlinson, M. Woolgar, M. Murray, L., Molteno, C. (1999)	Interview Structured Interview for DSM -IV (SCID)	R:socioeconomic status R:social support R:post-partum depression	Presence of a helpful partner was associated with better maternal sensitivity. No support from partner resulted in higher rates of PPD.
5. McMahan True, M., Pisani, L. & Oumar, F. (2001)	Strange Situation (amended), Weigh-In Procedure	R:maternal frightened/frightening behaviours O:attachment	No relation between sensitivity and frightening/frightened behaviours. Sensitivity was not associated with secure attachment. Mothers of disorganised infants had significantly higher ratings of frightened or frightening behaviours. Attachment distribution: 67% secure, 0% avoidant, 8% resistant, and 25% disorganised.
6. Perez, E., Hendricks, M.K., Beard, J.L., Murray-Kolb, L.E., Berg, A., Tomlinson, M., Irlam, J., Isaacs, W., Njengele, T., Sive, A. & Vernon-Feagans, L. (2005)	Blood Sample Griffiths Scale of Infant Development	R:maternal iron deficiency R:social support O:child cognitive development	Infants with mothers who had good support from partner showed better verbal, responsiveness, play, teaching, control and negative statements (PCIS subscales) at 9 month follow up. The quality of the relationship to the mother’s partner correlated positively with 6 of the 9 aspects of mother-child interaction. Mothers with less income did not differ from those with a higher income on the PCIS scales. At follow up, control group infants performed better in locomotor and GQ scales than Infants of anaemic mothers (Fe and PL).
7. Tomlinson, M., Cooper, P.J. & Murray, L. (2005)	Structured Interview for DSM -IV (SCID) Interview Strange Situation at 18 months	R:post-partum depression R:social support O:attachment	Sensitivity at 2 months was not associated with attachment at 18 months. Sensitivity at 18 months was associated with attachment. Postpartum depression at 2 months, and indices of compromised parenting at both 2 and 18 months, were associated with insecure infant attachment. At 18 months: 61.9% of the infants rated as securely attached; 4.1% as avoidant; 8.2% as resistant; and 25.8% disorganised.
8. Murray- Kolb, L. & Beard, J.L. (2009)	Blood Sample	R:maternal iron deficiency	Women with iron deficiency anaemia were less emotionally available to infants and, in turn, the infants were less responsive to mothers.
9. Bozicevic, L., De Pascalis, L., Schuitmaker, N., Tomlinson, M., Cooper, P. & Murray, L. (2016)	Child emotion regulation – assessed on two dimensions – reactivity to frustration and ER strategies; and child aggression assessed using the part of the CBCL	O:child socioemotional development	Positive relationship between sensitivity and child passive gaze (ER strategy) in all 3 samples. Stellenbosch group: high maternal sensitivity was positively associated with both child distraction and self-soothing. Higher sensitivity associated with fewer attempts to obtain the toy in the Stellenbosch sample. UK children: higher maternal sensitivity was associated with more use of distraction by infant (ER strategy). Higher sensitivity was associated with lower levels of child avoidance in the UK sample. Khayelitsha: by contrast, high sensitivity was associated with lower levels of distraction and self-soothing.
10a) Dawson, N., Bain, K. & Mesman, J. (2018)	Parent Development Interview Sociodemographic information	R:reflective functioning R:maternal education R:maternal income R:infant age and gender	No significant association between measure (Ainsworth sensitivity) and reflective functioning suggesting that both concepts remain discreet. Maternal educational level significantly positively associated with sensitivity scores for MBQS mini.
10b) Dawson, N., Bain, K. & Mesman, J. (2021)	Sociodemographic information	R:maternal education R:maternal employment status R:maternal age R:infant age R:infant gender	Maternal education was associated with maternal sensitivity only on the original MBQS but not on the SA Revised version.
Intervention with Maternal Sensitivity as variable			
11. Cooper, P.J., Landman, M., Tomlinson, M., Molteno, C., Swartz, L. & Murray, L. (2002)	Structured Interview for DSM-IV (SCID)	R:post-partum depression O:infant physical growth	Positively changed sensitivity did not positively influence maternal mood. Positively changed sensitivity in intervention group infants showed better growth than control group infants.
12. Klein, P. & Rye, H. (2004)	The Rutter Scale of Emotional and Social Development The Communicative Development Inventory	O:child socioemotional development O:child cognitive development	No statistically significant effect was found for the intervention’s impact on socio-emotional development – however, descriptively, children were rated as less hostile, aggressive, anxious, and hyperactive/distractible. Significant differences were found in language development between the intervention (improved) and control group.
13. Cooper, P.J., Tomlinson, M., Swartz, L., Landman, M., Molteno, C., Stein, A., McPherson, K. & Murray, L. (2009)	T3 – Strange Situation	R:social support R:post-partum depression O:attachment	No significant association between infant attachment status and parenting variables (sensitivity and intrusiveness). Although trending towards a positive association between improved sensitivity and secure attachment the mean sensitivity ratings at 12 months for secure versus insecure infants were not significantly different. No significant correlation was found between sensitivity/intrusiveness and depressive disorder.
14. Bigelow, A., Littlejohn, M., Bergman, N. & McDonald, C. (2010)	Sociodemographic information In hospital SSC education Follow up home visits by nurses	R:skin to skin contact (SSC) R:maternal education R:employment Status R:infant gestational age	Scores on the Maternal Behaviour subscale of the NCATS were significant and positively correlated with maternal education. Total scores on the NCATS were significantly correlated with maternal education and maternal age. Higher educated mothers with more stable income status and older newborn infants were better able to sensitively attune to their infant with the addition of SSC into their routine.
15. Cooper, P., Vally, Z., Cooper, H., Radford, T., Sharples, A., Tomlinson, M. & Murray, L. (2013)	Modified Goldsmith and Rothbart assessment protocol - 1 = unfocused, undirected behaviour, 2 = minimal, casual attention (e.g. holding or touching the toy with no focus),3 = clear focus, but not purposeful play, 4 = focused purposeful play (e.g. creating a pile of blocks) A series of picture cards were selected to present to infants- determined through a focus group.	O:child cognitive development (attention and language)	Researchers report that improved sensitivity (book-sharing tasks and play), facilitation and reciprocity (book-sharing only) post-training were related to infants’ improved comprehension and vocabulary – however, no significant statistics have been reported in the paper.
16. Murray, L., De Pascalis, L., Tomlinson, M., Vally, Z., Dadomo, H., MacLachlan, B., Woodward, C. & Cooper, P. (2016)	Child attention (rated low to high 1-5) Child vocalisations (rated through event counts)	O:child cognitive development (CDI-U; PPVT-R; ECVT) O:child socioemotional development (The Help Task; social interaction imitation - only assessed at follow-up)	Compared to the control group, intervention group carers showed greater sensitivity during the follow-up assessment during book sharing. Sensitivity during book sharing significantly predicted PPVT-R performance. Improvement in carer–child reciprocity during book sharing significantly predicted CDI-U scores and ECVT scores. In terms of prosocial behaviour, children in the intervention group showed more helping than those in the control group
17. Pitilas, C., Berastegui, A., Halty, A., Roderiguez, P., Kamara, M. & Mesman, J. (2020)	Parenting Stress Index Short Form 1^st^ 2 subscales Focus group to establish main concerns with resultant questionnaire. UNICEF Multiple Indicator Cluster Survey	R&O:parent stress R&O:child problems R&O:violent discipline	Even though a significant result was obtained for sensitivity post-intervention, it was not statistically associated with positive changes in the self-report variables (parenting stress, child problems, violent discipline).
18. Tomlinson, M., Rabie, S., Skeen, S., Hunt, X., Murray, L. & Cooper, P. (2020)	5 minute Observation (post hoc analysis of research conducted between 1999 and 2009)	R:maternal intrusiveness during feeding	Descriptively, maternal responsiveness to infant cues and synchronous interactions was higher among non-breastfeeding intervention mothers compared to control group mothers. Descriptively, intervention improved intrusiveness level in breastfeeding and non-breastfeeding mothers. Breast-feeding mothers were less intrusive than non-breastfeeding mothers in the control group.

*Note* R: Risks and O: Outcomes.

### Maternal sensitivity

Maternal sensitivity was broadly defined by authors as the mother’s ability to perceive and respond to various cues attending to her infant. See Table 2 and Table 3 for details. A number of points deserve emphasis. First, where sensitivity was measured as an independent variable through observation and characterised, even though infants were handled somewhat roughly at times, the authors reported that sensitivity was not completely compromised (Dixon *et al*, 1984; Mesman *et al*., 2018; Mesman *et al*., 2021). Secondly, in these African mothers, sensitivity was often expressed non-verbally (Broesch *et al*., 2016; Dawson *et al*., 2018, 2021; Mesman, Basweti, *et al.*, 2018; Mesman *et al*., 2021).

### Maternal sensitivity in relation to risks and outcomes

Studies that focused on risks to and outcomes (Table 3) suggested that maternal sensitivity and maternal mental health were compromised by a lack of social support (Tomlinson *et al*., 2005; Cooper *et al*., 2009). Poorer mothers with anaemia but adequate partner support were sensitive in their behaviours (Perez *et al*., 2005); although otherwise iron-deficient mothers were less sensitive and emotionally available than healthy control group mothers (Perez *et al*., 2005; Murray-Kolb & Beard, 2009). High maternal education levels did not always predict adequate sensitivity scores (Dawson *et al*., 2018, 2021). No clear evidence was presented to confirm western findings of sensitivity positively predicting secure attachment status (McMahan-True *et al*., 2001; Tomlinson *et al*., 2005) except in one sub-analysis of the Tomlinson *et al*. (2005) paper, where adequate concurrent sensitivity and secure attachment at 18 months was demonstrated. Sensitivity had a positive relationship with one passive infant emotion regulation technique (passive gaze) in three communities of varying SES (Bozicevic *et al*., 2016).

### Maternal sensitivity as a variable in intervention studies

In intervention studies (Table 3), improved sensitivity was associated with positive physical growth of infant (Cooper *et al*., 2002), child cognitive development (Klein & Rye, 2004; Cooper *et al*., 2013; Murray *et al*., 2016), and child socio-emotional development (Murray *et al*., 2016; Pitillas *et al*., 2021). Cooper *et al*. (2009) found that even with improved sensitivity and less intrusiveness there was no significant association between these maternal variables and attachment status.

### Sample SES

Across all reviewed studies, researchers focussed on samples from economically disadvantaged or rural populations, except where the Bozicevic *et al*. (2016) study sampled two contrasting SES populations in Cape Town, and two studies (Dixon *et al*., 1984; Broesch *et al*., 2016) included samples from the USA as part of their analysis.

The small number of studies, representing only five countries, and the limited risk and outcome factors examined, over short follow-up periods, must be noted. Given these cautions, the reviewed findings seem to suggest that maternal sensitivity and its effects, to the extent that they have been investigated, are consistent with what is demonstrated in western literature.

### Downs and black assessment tool

On the basis of the Downs and Black rating, the methods used in the reviewed papers appear satisfactory. Only one older paper was deemed fair, while all others were either classified as good or excellent. Despite this positive outcome, it should be noted that this assessment tool does not address whether cross-cultural factors have been considered during recruitment of sample, data collection, or analysis and interpretation of results. Factors relating to cross-cultural methodological rigour that were not considered in the Downs and Black methodological assessment tool can be referenced in Table 4.

**Table 4.** Cross-cultural research considerations in data collection and coding not assessed by downs and black rating

Formal translation of sensitivity measures/ questionnaires	Formal translation of risk (R) and outcome measures (O)	Validation of measures for context	Coding by appropriately trained first language speakers.	If not, were responses formally translated for coding by non-language-speaking coders.
Maternal Sensitivity Observed				
1. No	N/A	No	No	No
2. No	N/A	No	No	No
3a) No	N/A	No, but some adaptation	No	No
3b) No	N/A	No, but some adaptation	No	No
Maternal Sensitivity Observed in Relation to Risks and Outcomes				
4. No	R: Yes	No	Yes	No
5. No	R: Yes O: Yes	O: No, but some adaptation	No	No
6. No	R: Yes O: No	No	No	No
7. No	R: Yes O: No	No	No	No English-speaking South African 2nd author coded with a Xhosa-speaking research assistant.
8. No	R: N/A	No	No	No
9. No	O: No	No	No	No
10a) No	R: No	No, but some adaptation	No	No
10b) No	R: No	No, but some adaptation	No	No
Intervention with Maternal Sensitivity as variable				
11. No	R: Yes O: N/A	No	No	No
12. No	O: No	No, but some adaptation	No	No
13. No	R: Yes O: No	No, but some adaptation	No	No
14. No	N/A	No	No	No
15. No	O: PPVT Yes	O: No, but some adaptation	No	No
16. No	O: CDI Yes O: PPVT Yes	O: No, but some adaptation	No	No
17. No	R&O: Parent Stress Yes R&O: Child Problems Yes R&O: Violent discipline Yes	No, but some adaptation	No	No
18. No	R: No	No	No	No

*Note It is possible that in some of the research reviewed in this paper these considerations were made but not reported.

### Cross-cultural research considerations in data collection and coding

The information in Table 4 suggests that researchers reported limited implementation of important cross-cultural methodological considerations. There were no reports of formal translation of sensitivity measures or questionnaires, limited translation of risk and outcome measures, and critically, no formal validation of these measures for context, and no report of observational video translations for coding by researchers. The (McMahan-True *et al*., 2001) sensitivity coding was based on written records of the home observation videotape. No details about coders or translation have been provided. One study (Cooper *et al*., 1999) reported that an appropriately trained first language coder was used.

## Discussion

A total of 20 English language, peer-reviewed papers on maternal sensitivity were found, from only 5 of 55 Sub-Saharan countries. The majority (13 papers) stemmed from areas around two metro centres in South Africa (Cape Town and Johannesburg). Kenya produced four papers, and single studies are available for Mali, Ethiopia, and Sierra Leone. In addition, besides one Cape Town intervention study (Cooper *et al*., 2009), the sample sizes for the included studies are small. The lack of sufficient literature to represent this vast and diverse region is evident. This problematic dearth of knowledge production from Africa could be attributed to there being less available funding and fewer research groups to do the work (Jeenah & Pouris, 2008; Cooper & Nicholas, 2012). To date, most funding to the region has been allocated to the fields of malaria, HIV/AIDS, and tuberculosis research (Asante *et al*., 2020).

Few studies focussed entirely on maternal sensitivity (four papers). Eight focussed on risks to maternal sensitivity or child outcomes related to the construct; while another eight reported on interventions aimed at improving maternal sensitivity. The results of these limited investigations seem to indicate comparable effects to those reported in HICs (maternal education positively impacts sensitivity; poor support for mother has negative effect on sensitivity, interventions targeted at maternal sensitivity have positive impact) with the following possible exceptions: generally low sensitivity ratings in some studies; evidence of rough handling not received negatively by the child; sensitivity often expressed predominantly non-verbally; and the predictive association of sensitivity with secure attachment not clearly established.

Evaluation of studies using a standard, western developed quality assessment tool gave very positive results regarding research quality; however, detailed investigation of the extent to which central cross-cultural methodological principles were employed gave a different picture. Researchers either failed to report their methods fully or neglected to consider vital aspects of cross-cultural research (see Methodological Considerations below) – the review cannot establish which is the case. The following considerations were reported:

All but two studies (Murray-Kolb & Beard, 2009; Tomlinson *et al*., 2020) reportedly engaged local authorities or community members during recruitment and consent for research. Trained community members were reported to be employed to assist in five intervention studies (Cooper *et al*., 2002; Cooper *et al*., 2009; Cooper *et al*., 2013; Murray *et al*., 2016; Tomlinson *et al*., 2020). Two intervention studies (Klein & Rye, 2004; Pitillas *et al*., 2021) reported using local first-language paraprofessionals to conduct the research. Ten studies reported modified measures to accommodate the cultural nuances of the population.

As reflected in the focus of several reviewed studies, the primary reason maternal sensitivity is of interest because of its demonstrated effect on child outcomes, at least in western literature. It appears that the quantity and quality of focus on maternal input as it relates to child development in sub-Saharan Africa does not match that of western, educated, industrialised, rich, and democratic (WEIRD) countries (Henrich *et al*., 2010).

This dearth of peer-reviewed scientific psychological studies in sub-Saharan Africa since Ainsworth’s original work (Mesman *et al*., 2012; Mesman & Emmen, 2013) is disturbing because imported notions of child development and parenting potentially skew perceptions and risk impeding respect for people who do not fit criteria of HIC contexts (Nsamenang & Dawes, 1998; Henrich *et al*., 2010). Broesch *et al.* (2016) make an important point: The way western mothers respond to their infants may be unique when compared to majority of the world’s population which is in fact rural and non-westernised, arguing therefore that more research must be conducted to understand maternal responsiveness in contexts other than HICs (Broesch *et al*., 2016).

Also of concern is that to date, in sharp contrast to reported work in the (Deans, 2018) systematic literature review, an extremely limited set of risks (social support, maternal frightening behaviours, maternal iron deficiency, postpartum depression, reflective functioning, maternal education, parent stress, child problems) and outcomes (attachment status, child physical growth, language development, attention, emotion regulation strategies, and helping behaviour) has been examined. Given the number of challenges present across the continent, this seems inadequate. Of further concern is that the reviewed studies include only short-term follow up of one or two areas of function. The variability of child-rearing practices, the multiplicity of cultures, and the range of socio-economic and related psychosocial factors likely to impact on mothers and developing infants and toddlers across Sub-Saharan Africa have yet to be adequately explored and articulated.

Issues that have received scant consideration include the following: With the exception of the work conducted recently in Kenya by Mesman where alloparenting informed a reformulation of maternal sensitivity to “infant received sensitivity”, the role of extended family networks in sensitive caregiving is seldom considered. Extended family caregiving practices are common and valued in non-western contexts and are pivotal to the well-being of family systems largely unlike the primary dyad and nuclear family units in individualistic western contexts. Research from other LMICs suggests this may constitute a resilience factor (Fourment *et al*., 2018) and warrants more attention in African studies in the progress towards decolonising psychology on the continent. This reality is key to building a knowledge base that is representative of the varied parenting context in Sub-Saharan Africa.

The differing roles mothers assume appear to vary across cultures, and this is not considered in the studies reviewed. Evidence from other LMICs is suggestive – for example, Valenzuela (1997) in Chile found that sensitivity in mothers functioning as caregivers, attachment figures, and teachers could be considered universal, while mother as playmate was a foreign concept. Similarly, mothers in Yemen were unfamiliar with interacting with their children (face-to-face interactive play) in the manner required for observation (Alsarhi *et al*., 2018). Face-to-face interaction is a key component when coding for sensitivity in several commonly used measures which may not be appropriate in non-western contexts (Mesman, 2018). The degree to which mothers are comfortable with being observed and video-recorded is seldom considered: only four studies (two of which were reanalysis in 2021) specifically reported that their participants reacted mostly with ease to being observed and videoed (Dawson *et al*., 2018, 2021; Mesman, Basweti, et al., 2018; Mesman *et al*., 2021). Notably, the role a child plays in the dynamic interaction of the dyad in Africa is crucial and has been neglected as a focus of study in relation to the quality of maternal characteristics.

## Methodological considerations

It would seem imperative that the method and interpretive gaze used when studying non-western samples considers and is sensitive to nuances of context. An important question when reading the literature reviewed above is whether or to what extent the theoretical perspectives and hence methods employed permit observation of divergence from high-income country models of ideal parenting.

### Quality assessment of papers

The Downs and Black quality assessment tool was used to rate each paper (Downs & Black, 1998; O'Connor *et al.*, 2015). Quality assessment of only one older paper was deemed fair, while all others were either classified as good or excellent. Despite this positive outcome, it should be noted that the assessment tool does not address whether cultural factors have been considered during the recruitment of sample, data collection or analysis, and interpretation of results. Meaningful cross-cultural research should include proper translation of measures, validation of measures for the particular context, and participant responses appropriately translated especially when coded by experts who are not first-language speakers. However in this review, we found only one study (Cooper *et al*., 1999) used a first language trained lay person for coding and no one translated videos for English speaking coders. Nine did some measure translation (Cooper *et al*., 1999; McMahan-True *et al*., 2001; Cooper *et al*., 2002; Perez *et al*., 2005; Tomlinson *et al*., 2005; Cooper *et al*., 2009; Cooper *et al*., 2013; Murray *et al*., 2016; Pitillas *et al*., 2021), and eight made slight adaptations to measures for context (McMahan-True *et al*., 2001; Klein & Rye, 2004; Cooper *et al*., 2009; Cooper *et al*., 2013; Dawson *et al*., 2018; McMahan-True *et al*., 2001; Mesman, Basweti, *et al.*, 2018; Murray *et al*., 2016; Mesman *et al*., 2021; Pitillas *et al*., 2021). In addition, establishing measure invariance would strengthen the quality of data (Beaton *et al*., 2000; Boer *et al*., 2018; Aival-Naveh *et al*., 2019).

### Employing local vs research/clinical experts

A primary consideration, increasingly evident to us in working with diverse populations in South Africa, centres on who does the research and what impact this may have on results. Many measures of maternal sensitivity and dyadic interaction require a high level of (often postgraduate) psychological training along with adequate clinical experience to administer and code reliably. However, in many contexts such expertise is extremely scarce, thus impeding contextually sensitive cross-cultural investigations. Researchers are often faced with a choice: employ lay research assistants with limited training, but with local language and community expertise versus highly qualified researchers who often have to work with translators. Each option has marked benefits and disadvantages. While those embedded in the community will have insight into norms and practices, hence providing the research team with an invaluable perspective, social desirability bias or perceived demand characteristics may impede objectivity rendering results unreliable. Where research/clinical experts are employed, we must recognise the power differentials such a research team entering a community invariably introduces. Moreover, highly trained coders may have a limited, theoretically determined gaze and may not recognise nuanced meaningful data, rendering them blind to the emergence of subtle differences not otherwise expected. There is much room for more emphasis and funding to develop local experts to engage in knowledge creation.

### Language considerations

Notably, even though it is relevant for coding of sensitivity and dyadic interactions, only one study reported utilising a first-language speaker to code sensitivity (Cooper *et al*., 1999) and one other was coded by a clinician with assistance from a first-language research assistant (Tomlinson *et al*., 2005) – the implications of this should be considered. When videos are not translated, coding relies on non-verbal behaviour. Three studies acknowledge this (Bozicevic *et al*., 2016; Mesman *et al*., 2021) when videos are coded solely on observations of non-verbal interactions, coders risk missing nuanced incongruences between verbal and non-verbal communication. On the other hand, as noted in the Malian study (McMahan-True *et al*., 2001) where coding solely occurred based on written reports of videoed interactions, non-verbal communication could have been missed. Furthermore, if videos are not translated and subtitled, language could be variably interpreted and therefore coded differently by different interpreters or coders.

### Concepts and limiting lenses

Most measures included in the review above have been developed in contexts that do not mirror LMIC environments and we must consider the potential of them providing misleading negative characterisations about behaviour, which can influence public opinion about cultures foreign to individualistic western developed ones. For example, anthropological studies of the Gusii in Kenya (between the 1960s and 1990s), who have complex cultural practices in relation to marriage affecting child rearing, missed nuanced maternal sensitive behaviours. The belief that Gusii mothers are highly insensitive in their caregiving has been refuted in subsequent observations (Mesman, Minter, *et al.*, 2018).

The papers reviewed gave some consideration to the appropriateness of their measures. For example, Cooper *et al*. (1999) noted that despite being developed in the UK their maternal sensitivity measure was reliable in the context of Khayelitsha. The measure was utilised in a variety of contexts where meaningful group differences emerged allaying concerns about the Khayelitsha sample’s very high prevalence of maternal insensitive behaviours. The authors concluded that even though the overall mean ratings were low, many mothers were rated as highly sensitive suggesting that the measure was sensitive to variations in the sample. However, the only researchers to formally investigate measures assessed the performance of two established measures of maternal sensitivity and cautioned against accepting sensitivity to variation as evidence of validity (Dawson *et al*., 2018, 2021). These authors found that the MBQS did result in some mothers being rated as highly sensitive; however, these profiles were not consistent with those obtained for the same sample using the Ainsworth Sensitivity Scale, with the latter resulting in a far larger proportion of interactions being coded as sensitive. The difficulty of being unable to capture something a tool is not designed to measure remains a critical issue with which researchers must engage. Damaging theories based on biased measures and interpretation have been dangerous in the past (Henrich *et al*., 2010; Mesman, Minter, *et al.*, 2018). Researchers have a social, psychological, and ethical responsibility to produce scientific knowledge that does not harm but rather advances understanding of the nuanced social and ecological context of a child.

Careful detailed attention must thus be paid to interpretation of behaviours in non-western samples as this can affect coding. The Ethiopian intervention study (Klein & Rye, 2004) was careful to use local cultural norms and values in relation to the cognitive and emotional needs of the child during parental sensitization. The Weigh-In measure in the Malian study is the only novel non-western measure developed for this context. This study also documented potential culture-specific frightened/frightening behaviour and its impact on maternal sensitivity. These authors (McMahan-True *et al*., 2001) deliberately accounted for the possibility that Dogon infants would show behaviour patterns not captured by traditional attachment classifications. Importantly, they were able to work in collaboration with a local psychiatrist well versed in mother–infant relations. More recently, Mesman has worked in close collaboration with local researchers in various contexts to address the need for nuanced knowledge of maternal sensitive behaviours (Alsarhi *et al*., 2018; Asanjarani *et al*., 2018; Dawson *et al*., 2018; Fourment *et al*., 2018; Fourment *et al*., 2018; Mesman, Basweti, *et al.*, 2018; Ribeiro-Accioly *et al*., 2018; Mesman *et al*., 2021; Pitillas *et al*., 2021). Sensitivity measures, such as the MBQS mini which set relatively rigid definitions for sensitive responding appropriate for WEIRD countries, are being challenged (Dawson *et al*., 2018, 2021; Mesman, Basweti, *et al.*, 2018; Mesman *et al*., 2021).

### Defining the difference between sensitivity and warmth

In Ainsworth’s original work on attachment and sensitivity in Uganda, it was clear that dyads were more securely attached, and a higher degree of sensitive behaviours were displayed in comparison to her Baltimore sample (MacDonald, 1992). MacDonald (1992) proposed that there is a difference between warmth-seeking as a positive social reward system versus security-seeking in attachment which is fear or anxiety motivated. Ugandan mothers may not have displayed warm behaviours in the form of hugging and kissing but were highly attuned to their infants needs and signals. Two general conceptions of infant care can be observed cross-culturally (Keller *et al*., 1999; Keller *et al*., 2018) – 1) fostering close proximity to primary caregivers through extended body contact via carrying and co-sleeping arrangements, with indirect dyadic interactions, seems prevalent in non-western environments and in contrast 2) the dominant concept of care in western environments comprises mostly direct dyadic and short-term face to face interactive episodes. Three studies reviewed here (Cooper *et al*., 1999; Tomlinson *et al*., 2005; Bozicevic *et al*., 2016) have reported that warmth was included in their maternal sensitivity measure. No further expansion on the particular role and effects of warmth was reported. In this review, only one study (Mesman *et al*., 2021) of the Gusii addresses warmth as a discreet separate component for discussion. Mesman notes that while less frequent than has been observed in western samples, behavioural expressions of warmth through hugging, kissing, smiling, and touch were present. There has been a tendency to generalise western notions and expectations of maternal competencies to the rest of the world. Assessing warmth as part of sensitivity is questionable when in some languages no words exist for parental warmth, love, and affection (Keller *et al*., 2018). As mentioned previously, this should be cautioned against and a nuanced understanding of the different components of maternal in/sensitivity should be allowed to emerge. Whether warmth is necessary to foster emotional and physical safety for the survival of the child has been questioned (MacDonald, 1992). A caregiver could display warm characteristics, yet attune inappropriately to the emotional and physical needs of their child. Potentially, differing parenting goals between a predominantly individualistic western reward-driven context and historically community-driven non-western rural societies could motivate divergent approaches to creating healthy bonds between caregiver and infant.

### Limitations

A conventional PRISMA compliant search method could not be used because a search using all the key search terms turned up no papers. For a yield of pertinent study data, six searches employing different combinations of the core foci were made. Highly heterogenous research foci and variable methods prohibited any direct or statistical comparison of findings and made it challenging to descriptively compare studies.

Sub-Saharan Africa has several Francophone countries, but French language papers were not considered for this review.

This review focussed on research using objective quantitative coding of observed maternal sensitivity. The use of maternal sensitivity exclusively rather than including non-gendered terms such as parental sensitivity (Broom, 1994; Bakermans-Kranenburg *et al*., 2003; Mesman & Emmen, 2013) as a search term can be considered a limitation; however, sensitivity research is still in its infancy in SSA. In very recent studies of family roles undertaken in South Africa (Hatch & Posel, 2018; Adebiyi *et al*., 2021), females are still primary caregivers. In contrast, maternal sensitivity research has been extensive in the global north, and the limited focus of sensitivity in relation to mother as primary caregiver has expanded its gaze to fathers as primary caregivers (West *et al*., 2009; Jones *et al*., 2022). SSA sensitivity research should explore if and how such expansion is applicable. In South Africa, for example, many children are raised by their grandmothers or are from female-headed single parent households (*Stats SA General Household Survey*, 2021). We note however that the exclusion of non-gendered search terms in our search may have resulted in some papers being missed in this review.

Additionally, although these were not included in our review, theoretical (critical literature reviews) and qualitative papers have added to the characterisation and reconceptualization of the concept in sub-Saharan Africa (Dawson, 2022).

## Conclusion

Maternal sensitivity is an important construct that was developed including seminal evidence from Africa. However, there is relatively little work on it since Ainsworth’s research in sub-Saharan Africa. The review highlights the limited research available, along with extremely limited knowledge regarding risk factors and child outcomes related to maternal sensitivity. Considering the importance of understanding sensitive caregiving in aid of fostering emotionally and physically healthy children, additional research in this area should be encouraged. The positive influence of reviewed interventions on maternal sensitivity, albeit small and modest, could be encouraging for further implementation. Critically, what has emerged from this review are some interesting and important reflections on the landscape of maternal sensitivity research. Fundamental methodological principles such as translation and validation of measures for non-western contexts must be implemented. Issues such as researcher gaze and its impact on the nature and quality of knowledge created must be taken into account. Researcher awareness of potentially unique caregiving systems and role of mother/caregiver within the sample must inform future responsible research design. It is hopeful that within the last 5 years nuanced sensitivity knowledge from LMICS has been generated (Alsarhi *et al*., 2018; Asanjarani *et al*., 2018; Fourment *et al*., 2018; Mesman, 2018; Ribeiro-Accioly *et al*., 2018), and we hope for significant growth and expansion as seen in the global north.
